# Supplementary material for: Structure of the AlgKX modification and secretion complex required for alginate production and biofilm attachment in Pseudomonas aeruginosa
Source: Nat Commun. 2022 Dec 9;13:7631. doi: 10.1038/s41467-022-35131-6 (PMC9734138; doi:10.1038/s41467-022-35131-6)
Supplement: Supplementary file 1 — Supplementary information [file 41467_2022_35131_MOESM1_ESM.pdf]

Supplementary Information for

Structure of the AlgKX modification and secretion complex required for alginate production and biofilm attachment in *Pseudomonas aeruginosa*

Andreea A. Gheorghita[1,2], Yancheng Evelyn Li[1,2,$], Elena N. Kitova[3], Duong T. Bui[3], Roland Pfoh[1], Kristin E. Low[1,%], Gregory B. Whitfield[1,2,^], Marthe T. C. Walvoort[4,#], Qingju Zhang[4,&], Jeroen D. C. Codée[4], John S. Klassen[3], P. Lynne Howell[1,2,*]

From the [1]Program in Molecular Medicine, The Hospital for Sick Children, Toronto, Ontario, Canada; [2]Department of Biochemistry, University of Toronto, Toronto, Ontario, Canada; [3]Department of Chemistry, University of Alberta, Edmonton, Alberta, Canada [4]Leiden Institute of Chemistry, Leiden University, Leiden, The Netherlands

Current addresses:
[$]Division of Chemistry and Chemical Engineering, California Institute of Technology, Pasadena, California, USA
[%]Lethbridge Research and Development Centre, Agriculture and Agri-Food Canada, Lethbridge, Alberta, Canada
[^]Département de Microbiologie, Infectiologie et Immunologie, Université de Montréal, Montréal, Quebec, Canada
[#]Department of Chemical Biology, Stratingh Institute for Chemistry, University of Groningen, Groningen, The Netherlands
[&]National Research Centre for Carbohydrate Synthesis, Jiangxi Normal University, Nanchang, China

*Corresponding author: P. Lynne Howell
E-mail: howell@sickkids.ca

Running title: Structure of a modification and secretion complex required for biofilm production

Keywords: *Pseudomonas aeruginosa*, exopolysaccharide secretion system, biofilm, bacterial genetics, crystallography, structure-function

Supplementary Table 1: AlgKX data collection and refinement statistics.

	AlgKX[a] PDB: 7ULA
**Data collection**	
Space group	*I* 4 2 2
Cell dimensions	
a, b, c (Å)	169.95, 169.95, 143.02
α, β, γ (°)	90, 90, 90
Resolution (Å)	45.9-2.46 (2.55-2.46)[b]
Unique reflections	38142 (3741)
R_{merge}	0.063 (1.158)
$I/\sigma I$	33.2 (1.8)
Completeness (%)	100 (100)
Redundancy	13.9 (9.0)
**Refinement**	
Resolution (Å)	45.9-2.46
Reflections used	38104 (3715)
R_{work} / R_{free} (%)	22.1/25.8
No. atoms	
Protein	5644
Ligand/ion	31
Water	27
B-factors	
Protein	71.51
Ligand/ion	86.57
Water	58.90
R.m.s. deviations	
Bond lengths (Å)	0.005
Bond angles (°)	0.87
Ramachandran plot[c]	
Total favoured (%)	97.15
Total allowed (%)	2.72

[a]Data are from one crystal.
[b]Values in parentheses are for highest-resolution shell.
[c]As calculated by MolProbity[1].

Supplementary Table 2: Bacterial strains and plasmids used.

Strain	Description	Reference
E. coli		
DH5α	Cloning strain; F^- Φ80*lacZ* ΔM15 Δ(*lacZYA-argF*) U169 *recA1 endA1 hsdR17* (r_K^-, m_K^-) *phoA supE44* λ^- *thi*-1 *gyrA96 relA1*	Invitrogen
TOP10	Cloning strain: F^- *mcrA*Δ(*mrr-hsdRMS-mcrBC*) Φ80*lacZ* ΔM15 Δ*lacX74* Δ*araD139*Δ(*ara leu*)7697 *galU galK rpsL endA*1 *nupG*, Str^R	Invitrogen
SM10(λpir)	Biparental mating donor strain; *thi thr leu tonA labY supE recA*::RP4-2-Tc::Mu Km λpir, Kan^R	2
Lemo21(DE3)	*fhuA2 [lon] ompT gal* (λ DE3) *[dcm]* Δ*hsdS/ pLemo*(Cam^R) *λ DE3 = λ sBamHIo ΔEcoRI-B int::(lacI::PlacUV5::T7 gene1) i21 Δnin5* pLemo = pACYC184-*PrhaBAD-lysY*	New England BioLabs
BL21-CodonPlus(DE3)	*E. coli* B F^- *ompT hsdS*(r_B^- m_B^-) dcm^+ Tet^R *gal* λ(DE3) *endA* Hte [*argU proL* Cam^R] *[argU ileY leuW* Strep/$Spec^R$]	Agilent
P. aeruginosa		
FRD462	A chemically mutagenized strain of *P. aeruginosa* FRD1 that is incapable of incorporating guluronate residues into alginate, *algG4.*	3
FRD1	Mucoid alginate producing strain of *P. aeruginosa* isolated from the sputum of an individual with cystic fibrosis.	4
PAO1	Wild-type strain	M. R. Parsek
PAO1 Δ*wspF* P_{BAD}*alg*	PAO1 Δ*wspF* (in-frame); *araC*-P_{BAD} inserted upstream of *alg* operon	5
PAO1 Δ*wspF* P_{BAD}*alg* Gm^R	PAO1 Δ*wspF* (in-frame); *araC*-P_{BAD} inserted upstream of *alg* operon, Gen^R	6
GBW17	PAO1 Δ*wspF* P_{BAD}*alg* Δ*algX*	This study
GBW18	PAO1 Δ*wspF* P_{BAD}*alg* Δ*algX*, Gen^R	This study
GBW19	GBW17 *attTn7*::miniTn7T-Gm::*araC*-P_{BAD}AlgX$_{Pa}$, Gen^R	This study
GBW20	GBW17 *attTn7*::miniTn7T-Gm::*araC*-P_{BAD}AlgX$_{Pa}$-VSV-G, Gen^R	This study
GBW21	PAO1 Δ*wspF* P_{BAD}*alg* Δ*algK*	This study
GBW22	PAO1 Δ*wspF* P_{BAD}*alg* Δ*algK*, Gen^R	This study
GBW23	GBW21 *attTn7*::miniTn7T-Gm::*araC*-P_{BAD}AlgK$_{Pa}$, Gen^R	This study
GBW24	GBW21 *attTn7*::miniTn7T-Gm::*araC*-P_{BAD}AlgK$_{Pa}$-VSV-G, Gen^R	This study
GBW25	PAO1 Δ*wspF* P_{BAD}*alg* Δ*algG*	This study

GBW26	PAO1 Δ*wspF* P_{BAD}*alg* Δ*algG*, Gen^R	This study
GBW27	GBW25 *attTn7*::miniTn7T-Gm::*araC*-$P_{BAD}$$AlgG_{Pa}$, Gen^R	This study
GBW28	GBW25 *attTn7*::miniTn7T-Gm::*araC*-$P_{BAD}$$AlgG_{Pa}$-VSV-G, Gen^R	This study
**Plasmid**	**Description**	**Reference**
**Recombinant protein expression**		
pET26b	IPTG-inducible expression vector containing a C-terminal His6 tag, N-terminal PelB sequence, Kan^R	Novagen
pET28b	IPTG-inducible expression vector containing a N- and C-terminal His6 tag, N-terminal thrombin cleavage tag, Kan^R	Novagen
pET26b::$AlgK^{1\text{-}484}_{Pp}$	pET26b with *P. putida* KT2440 $AlgK^{1\text{-}484}$ (codon optimized by BioBasic for expression in *E. coli*) fused to a C-terminal His6 tag, Kan^R	This study
pET28a::$AlgK^{1\text{-}475}_{Pa}$	pET28a with *P. aeruginosa* PAO1 $AlgK^{1\text{-}475}$ fused to a C-terminal His6 tag, Kan^R (for antibody production)	This study
pET26b::$AlgK^{33\text{-}484}_{Pp}$	pET26b::$AlgK^{1\text{-}484}_{Pp}$ with signal sequence residues 1-32 deleted	This study
pET26b::$AlgX^{1\text{-}479}_{Pp}$	pET24b with *P. putida* KT2440 $AlgX^{1\text{-}479}$ (codon optimized by BioBasic for expression in *E. coli*) fused to a C-terminal His6 tag, Kan^R	This study
pET26b::$AlgX^{\Delta Nterm}_{Pp}$	pET24b::$AlgX^{1\text{-}479}_{Pp}$ with residues N-terminal region residues 1-38 deleted	This study
**Allelic exchange**		
pEX18Gm	Suicide vector for allelic exchange in *P. aeruginosa*, encodes SacB, Gen^R	7
pEX18Gm::Δ*algX*	pEX18Gm with *P. aeruginosa* PAO1 *algX* cloned between EcoRI and HindII sites, Gen^R	This study
pEX18Gm::Δ*algK*	pEX18Gm with *P. aeruginosa* PAO1 *algK* cloned between EcoRI and HindII sites, Gen^R	This study
pEX18Gm::Δ*algG*	pEX18Gm with *P. aeruginosa* PAO1 *algG* cloned between EcoRI and HindII sites, Gen^R	This study
**Complementation analysis**		
pJJH187	GateWay-compatible plasmid with the *araC* repressor and the P_{BAD} promoter flanked by attL1 and attR5 recombination sites; Km^R	8
pUC18T-miniTn7T-Gm	*aacC1* on miniTn7-based vector with transcriptional terminators at the right end of the Tn7 transposon; Amp^R, Gen^R	9
pUC18T-miniTn7T-Gm-pBAD	pUC18T-miniTn7T-Gm containing *araC*-P_{BAD} and a downstream MSC (SmaI-NotI-PstI-NcoI) cloned between the HindIII and SacI sites; Amp^R, Gen^R	5
pUC18T-miniTn7T-$AlgK_{Pa}$	pUC18T-miniTn7T-Gm-pBAD with *P. aeruginosa* PAO1 *algK* fused to an upstream synthetic ribosome	This study

	binding site, cloned between the NotI and NcoI sites, Gen^R	
pUC18T-miniTn7T-AlgK$_{VSV\text{-}G\ Pa}$	pUC18T-miniTn7T-AlgK$_{Pa}$ C-terminally tagged with VSV-G	This study
pUC18T-miniTn7T-AlgX$_{Pa}$	pUC18T-miniTn7T-Gm-pBAD with *P. aeruginosa* PAO1 *algX* fused to an upstream synthetic ribosome binding site, cloned between the PstI and SacI sites, Gen^R	This study
pUC18T-miniTn7T-AlgX$_{VSV\text{-}G\ Pa}$	pUC18T-miniTn7T-AlgX$_{Pa}$ C-terminally tagged with VSV-G	This study
pUC18T-miniTn7T-AlgX$^{\Delta Nterm}_{Pa}$	pUC18T-miniTn7T-Gm-pBAD with *P. aeruginosa* PAO1 AlgX residues 29-41 deleted fused to an upstream synthetic ribosome binding site, cloned between the PstI and SacI sites, Gen^R	This study
pUC18T-miniTn7T-AlgG$_{Pa}$	pUC18T-miniTn7T-Gm-pBAD with *P. aeruginosa* PAO1 *algG* fused to an upstream synthetic ribosome binding site, cloned between the PstI and SacI sites, Gen^R	This study
pUC18T-miniTn7T-AlgG$_{VSV\text{-}G\ Pa}$	pUC18T-miniTn7T-AlgG$_{Pa}$ C-terminally tagged with VSV-G	This study
pTNS2	Helper plasmid encoding *tnsABCD*, Amp^R	9

Supplementary Table 3: Primers used in this study.

Name	Sequence (5' → 3')
**Generation of *P. aeruginosa algK* chromosomal deletion**	
algKPA01upF	CGAGAGCTCCTCTTCGGCCACCAGATGAA
algKPA01upR	TCATAGGCTTTCTGGCTCTTCCAGGGGAGGGAGGATGGGCAT
algKPA01downF	GAGAGCCAGAAAGCCTATGAA
algKPA01downR	GAGAAGCTTTCGCCGGGGTAGGCGGTGAG
algKPA01SEQ-F	CCTTCTCGGCCAACCTGCTG
algKPA01SEQ-R	CGATGTTGGTGTCCTGCCAC
**Generation of *P. aeruginosa algX* chromosomal deletion**	
algXPA01upF	GGGGAATTCCTGCTCTGGGGCAACCAGG
algXPA01upR	TTACCTCCCGGCCACCGACTGGCGGAACAGTCGGGAAGTGCG
algXPA01downF	CAGTCGGTGGCCGGGAGGTAA
algXPA01downR	CCGAAGCTTGCCGGTGTGGTTGAAGTCGT
algXPA01SEQ-F	GCCTACAACGAGGTCTATCG
algXPA01SEQ-R	CCGCGAGCTGGAGAACTTCA
**Generation of *P. aeruginosa algG* chromosomal deletion**	
algGPA01upF	GGGGAATTCTTCACCGGCACCCGCTCGCG
algGPA01upR	TCAGTCCTGGAGTTCGGCCTGCGAAAGGGAAATGTCGGGCAT
algGPA01downF	CAGGCCGAACTCCAGGACTGA
algGPA01downR	GAGAAGCTTTCGCCAGCTCGTAGTCGAAG
algGPA01SEQ-F	CAGCGGCGGTGGCAAGGACG

algGPA01SEQ-R	GGCGAGAAGTCCGGGGTCCA
**Generation of AlgK in mini-Tn7 vector**	
algK_miniTn7_NotI	TGCGCGGCCGCGAGGAGGATATTCATGAAGATGCCCATCCTCCCTC
algK_miniTn7_NcoI	AGGCCATGGTCATAGGCTTTCTGGCTCTTCTTCG
**Generation of AlgK-VSV-g in mini-Tn7 vector**	
AlgK_Tn7_C-VSV-G-F	ATTCATTTCAATATCTGTACATAGGCTTTCTGGCTCTTCTTCGTTGATCG
AlgK_Tn7_C-VSV-G-R	AGATTAGGAAAATGACCATGGGAGCTCATGCATGATCGAATT
**Generation of AlgX in mini-Tn7 vector**	
algX_miniTn7_PstI	TATCTGCAGGAGGAGGATATTCATGAAAACCCGCACTTCCCGACTG
algX_miniTn7_SacI	ACTGAGCTCTTACCTCCCGGCCACCGACTGGCT
**Generation of AlgX-VSV-G in mini-Tn7 vector**	
AlgX_Tn7_C-VSV-G-R	ATTCATTTCAATATCTGTATACCTCCCGGCCACCGAC
AlgX_Tn7_C-VSV-G-F	AGATTAGGAAAATAAGAGCTCATGCATGATCGAATTA
**Generation of ΔN-term AlgX in mini-Tn7 vector**	
ΔN_term_cleavage_site	CGGCCGGGCACAGGTTGTCGGCGGCCAGGGCTGCCTGGGC
ΔN_term_2	AACCTGTGCCCGGCCGCCGCCTACGA
**Generation of AlgG in mini-Tn7 vector**	
algG-miniTn7_NotI	TATGCGGCCGCGAGGAGGATATTCATGCCCGACATTTCCCTTTCGAT
algG-miniTn7_NcoI	ACGCCATGGTCAGTCCTGGAGTTCGGCCTGGC
**Generation of AlgG-VSV-G in mini-Tn7 vector**	
AlgG_Tn7_C-VSV-G-F	ATAGATTAGGAAAATAACCATGGGAGCTC
AlgG_Tn7_C-VSV-G-R	TCATTCAATATCTCGTATAGTCCTGGAGTTC
**Generation of AlgX$^{\Delta Nterm}$ in pET24b**	
AlgXPp-ΔNterm-F	GCGCCGAAGACGTCCAGTTGTGCCCTGAAGCGC
AlgXPp-ΔNterm-R	GCGCCGAAGACGTCCAGTTGTGCCCTGAAGCGC
**Generation of AlgK$^{33\text{-}484}$ in pET26b**	
PpK5D33_NdeI	CGCCATATGTGTGCAGGTCTGCCGGATCAGC
PpK3_XhoI	TTAACTCGAGCAGGCTATCTTCGCCGTCAACTTCTTT
**Sequencing**	
miniTn7 Seq_F	GCGGATCCTAACTGACGCTT
miniTn7 Seq_R	CAAAGGGAATCAGGGATCTTGAAG

```
P. aeruginosa      1 MKMPILPPLPLASRHLL--------------LASAIALAAGCAGLPDQRL     36
                             :....|.::              ||:||.| |||||||||||
P. putida          1 --------MACEGRTMISDTYKVRVNLGLCALAAAITL-AGCAGLPDQRL     41

P. aeruginosa     37 AQEALERGDLATAQSNYQALAAMGYADAQVGLADMQVASGDSAQQAKAEK     86
                     |.|||:|||.|.|:.||:|||.:||::|||||||::||:.|.:|..:||.
P. putida         42 ANEALKRGDTALAERNYKALADLGYSEAQVGLADIKVATRDPSQIKEAEA     91

P. aeruginosa     87 LYREAAQTSPRARARLGKWLAAKPGASDAEHREAERLLSQAFEQGEDSAL    136
                     .||.||.|||||:||||:.|.|||.::.||..|||.||.||.:||:.:.|
P. putida         92 TYRAAAATSPRAQARLGRLLVAKPDSTQAEREEAETLLKQAAKQGQSNTL    141

P. aeruginosa    137 VPLIVLYLQYPQSWPEIDPQQRIDQWRARGLPQADLAQIILYRTQGTYAQ    186
                     :||.:|||.||||:|:::.||:||||||.|.|:|.|||::||||||||.|
P. putida        142 IPLAMLYLSYPQSFPKVNAQQQIDQWRAAGNPEAGLAQVLLYRTQGTYDQ    191

P. aeruginosa    187 HLGEIEQVCQRWLRRMDVCWYELATVYQMQGNAEKQKVLLEQLRAAYKAG    236
                     ||||:|::|:..|...|:|:.|||||||.:|.|::|..||.||::||..|
P. putida        192 HLGEVEKICKAALNTTDICYVELATVYQKRGQADQQAALLGQLKSAYARG    241

P. aeruginosa    237 RVPGERVDSVAGVLADGELGQPDPQTAQALLEEIAPSYPAAWVSLAKLLY    286
                     .||..||||||.||||..|||.|.:||:.|||::||:.||:|||||:|:|
P. putida        242 AVPATRVDSVARVLADRSLGQTDEKTAKELLEQVAPANPASWVSLAQLVY    291

P. aeruginosa    287 DYPDQGDLEKMLGYLKNAQDAAQPRAELLLGRLYYDGKWAPQDPRKAERH    336
                     |:|:.||.::::.|:...::|.|||||||||||||:||..|.|.:|||:|
P. putida        292 DFPELGDTDQLMAYIDKGREAEQPRAELLLGRLYYEGKTLPADAQKAEQH    341

P. aeruginosa    337 LLKAA-ASEPQANYYLGQIYRRGFLGKVYPQKAVDHLILAARAGQASADM    385
                     |..|| |.|..|:|||||:||||:||.|.||||||||:.|||.||.|||.
P. putida        342 LQAAAEAGEISAHYYLGQLYRRGYLGNVEPQKAVDHLLAAARGGQNSADY    391

P. aeruginosa    386 ALAQLWSQGRGIQPNRVNAYVFGQLAVQQQVPQASDLLGQIEAQLPPAER    435
                     |||||:|:|.||:|...||:||.||:.....||:::||.|::.||.|.:|
P. putida        392 ALAQLFSEGHGIRPQPGNAWVFAQLSQANPTPQSAELLQQLDQQLTPDQR    441

P. aeruginosa    436 SQAQQLLKREQQSRGNNWQ---ATVSLLQSQDSPINEEEPESL    475
                     :||||||.:|:::||:..|   :|::|...||.....:..:||
P. putida        442 NQAQQLLDQEKRARGSLAQGANSTLALEALQDDEKEVDGEDSL    484
```

Supplementary Figure 1: Sequence alignment of AlgK from *Pseudomonas aeruginosa* and *Pseudomonas putida*. Sequence alignment reveals the protein sequences share 54.4 % identity and 72.4 % similarity across the two species. | indicates positions which have a fully conserved residue; : indicates conservation between residues with strongly similar properties; . indicates conservation between residues with weakly similar properties/no similar properties; - indicates a gap in the sequence.

```
P. aeruginosa      1 MKTRTSRLFRLSALAAGLCLAQAALAADPGAAPSYQALPAGNLCPAAAYD     50
                        .|..|.:|..|:|.|......:.|:...||::.|.|...|||.|...
P. putida          1 ---MTPHLMKLLGLSAALLAISQGVRAEDVKAPTFSAEPCCQLCPEAHDA     47

P. aeruginosa     51 SRYNTKYLGFFTHLVQAQDDWLFRTTYDLRTDFGTSAEGWRELRALRDEL    100
                     |||.|:|...||.|||||.||||||..||||:|.|:..|::.|:.:.|..
P. putida         48 SRYTTRYQQNFTTLVQAQGDWLFRTREDLRTEFNTTPAGYKRLQQVHDAF     97

P. aeruginosa    101 KRKGIELVVVYQPTRGLVNREKLSPAEKAGFDYELAKKNYLATIARFRQA    150
                     |::|:|||||||||||||||..|:|||||.|||:.|..||.|.:.||...
P. putida         98 KKRGVELVVVYQPTRGLVNRNMLNPAEKAAFDYQKALGNYQAMLKRFASM    147

P. aeruginosa    151 GIWTPDFSPLFDEKEEHA-----YYFKGDHHWTPHGARRSAKIVAETLKQ    195
                     |...||.|||.:|:...|     :||:||.||||:||.|:|||||:|:.:
P. putida        148 GYNVPDLSPLTNEQLAAADQGKDFYFRGDQHWTPYGAERAAKIVADTVHK    197

P. aeruginosa    196 VPGFEEIPKKQFESKRVGLLSKLGTFHKAAAQLCGNSYATQYVDRFETEP    245
                     :|.||.||:|:||:::.|.:.|.||.|..|.||||.|||.||:|:|.|||
P. putida        198 MPAFEGIPRKEFETRKSGRMGKTGTLHNVAGQLCGTSYAVQYMDQFATEP    247

P. aeruginosa    246 VGASDSGDLFGDGGNPQIALVGTSNSGPAYNFAGFLEEFSGADILNNAVS    295
                     .|||...|||||.||.||.|||||:||..|||:||||::.|||:||.|..
P. putida        248 KGASGGDDLFGDSGNAQITLVGTSHSGKNYNFSGFLEQYIGADVLNVAFP    297

P. aeruginosa    296 GGGFDSSLLAYMTSEEFHKNPPKILIWEFATHYDMAQKSFYRQAMPLVDN    345
                     |||.:.|::.|:.||||.|||||||||||:..|.:.|::.:||.:.|:|:
P. putida        298 GGGLEGSMIQYLGSEEFQKNPPKILIWEFSPLYRLDQETIWRQILGLLDD    347

P. aeruginosa    346 GCSGRKTVLSRKVKLRQGRNEVLLNSAALPIR---SGSYVADVTYSDPSV    392
                     ||..|..::|....|:.|:||:::|.....|:   :.:...||.:.||||
P. putida        348 GCDDRPALMSASTTLKPGKNELMVNGKGGVIKDLINRNLQMDVKFEDPSV    397

P. aeruginosa    393 HELKNTIWYMNGRREQLKIEQSKAVDTGGRYVFQLRNDSDWADQQFLSLE    442
                     ..|:.|:||:|||.|.:|:|:.:..||.||:|||:|.|.|||.|:.|:.|
P. putida        398 KVLQATLWYLNGRHEDIKLEKPETSDTDGRFVFQMREDEDWASQRLLAFE    447

P. aeruginosa    443 IEAPEDMPQGLEVQASICQ----AAPAKASQSVAGR    474
                     ::.||...|  :|:|.:|:    |.||:.:|  ||:
P. putida        448 VQGPESGTQ--KVEAKLCKRNNFAVPAQTAQ--AGQ    479
```

Supplementary Figure 2: Sequence alignment of AlgX from *Pseudomonas aeruginosa* and *Pseudomonas putida*. Sequence alignment reveals the protein sequences share 49.0 % identity and 66.5 % similarity across the two species. | indicates positions which have a fully conserved residue; : indicates conservation between residues with strongly similar properties; . indicates conservation between residues with weakly similar properties/no similar properties; - indicates a gap in the sequence.

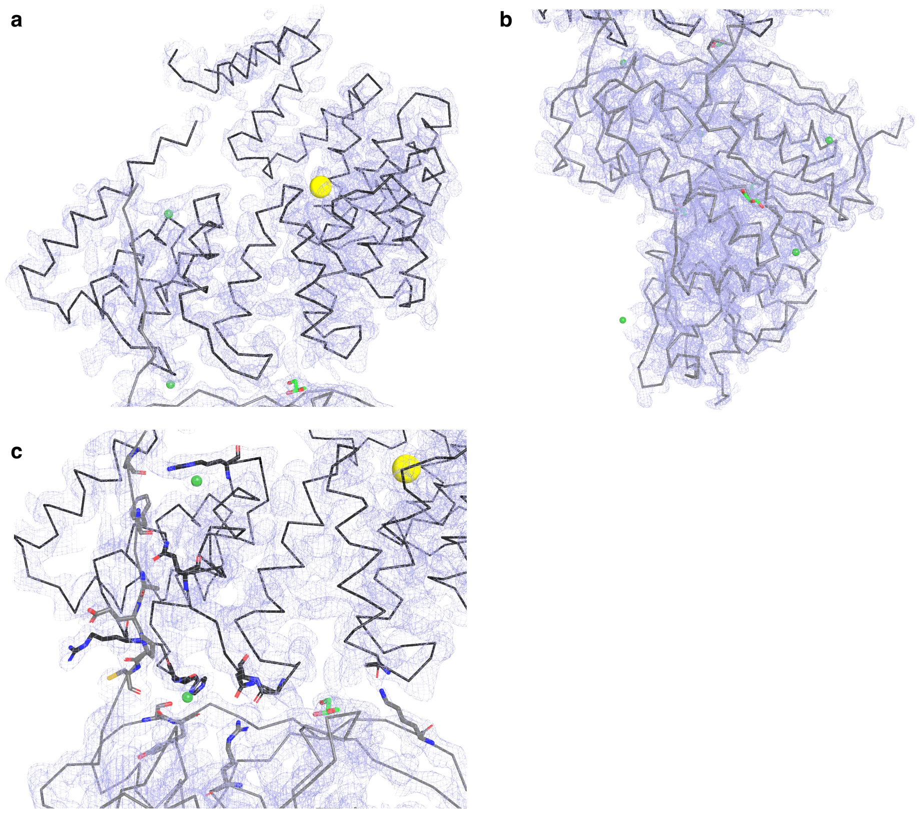

Supplementary Figure 3: Representative electron density. Simulated-annealing composite omit $2F_o$-F_c electron density maps contoured at 1σ and carved 1.6 Å from the model, with AlgK$_{Pp}$ and AlgX$_{Pp}$ coloured black and grey, respectively. **a**, Ribbon representation of AlgK$_{Pp}$. **b**, Ribbon representation of AlgX$_{Pp}$. **c**, View of the AlgKX$_{Pp}$ interaction interface.

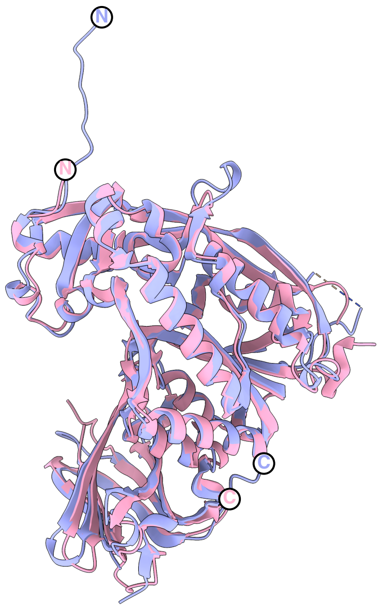

Supplementary Figure 4: The N-terminus of AlgX is stabilized in the AlgKX complex. Superimposition of the isolated AlgX structure from *P. aeruginosa* (pink) (PDB: 4KNC) and the complexed structure from *P. putida* (periwinkle) reveals a C_α RMSD of 0.778 Å.

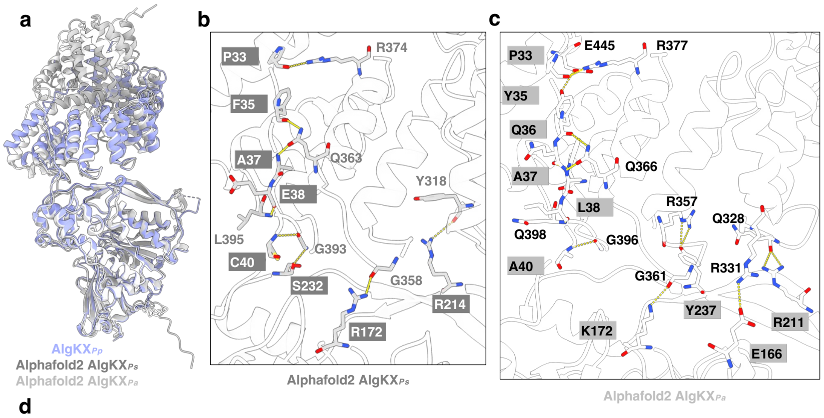

d

AlgX

```
P. putida        ---MTPHLMKLLGLS---AALLAISQGVRAEDVKAPTFSAEPCCQLCPEAHDASRYTTRY   54
P. syringae      ---MHAHLIKLLSLSSLTAALMAAAGVARADDAQAPTFKAEPCCSLCPAAHDAKNYTTRY   57
P. aeruginosa    ---MKTRTSRLFRLSALAAGLCLAQAALAADPGAAPSYQALPAGNLCPAAAYDSRYNTKY   57

P. putida        VNRNMLNPAEKAAFDYQKALGNYQAMLKRFASMGYNVPDLSPLTNEQLAAADQGKDFYFR  174
P. syringae      VDRNKLFPAERDKFDYDKALKNYQAMLGRFSKMGYWVPDLSPLTNEQ-----QAHDFYFR  172
P. aeruginosa    VNREKLSPAEKAGFDYELAKKNYLATIARFRQAGIWTPDFSPLFDEKE-----EHAYYFK  172

P. putida        GDQHWTPYGAERAAKIVADTVHKMPAFEGIPRKEFETRKSGRMGKTGTLHNVAGQLCGTS  234
P. syringae      GDQHWTPYGAQRTAKIVAETVKKVPGYSSIPKREFESHISGRMGKTGTLHNMAGQLCGTS  232
P. aeruginosa    GDHHWTPHGARRSAKIVAETLKQVPGFEEIPKKQFESKRVGLLSKLGTFHKAAAQLCGNS  232

P. putida        YAVQYMDQFATEPKGASGGDDLFGDSGNAQITLVGTSHSGKNYNFSGFLEQYIGADVLNV  294
P. syringae      YAVQYMDQFTTEPKGEAGDGDLFGDAGNPEITLVGTSHSGKNYNFAGFLQEYMGADVLNV  292
P. aeruginosa    YATQYVDRFETEPVGASDSGDLFGDGGNPQIALVGTSNSGPAYNFAGFLEEFSGADILNN  292
```

e

AlgK

```
P. putida        LGDTDQ---LMAYIDKGRE---------AEQ---PRAELLLGRLYYEGKTLPADAQKAEQ  340
P. syringae      LGDVNK---MMEYLNNGRA---------ADQ---PRAELLLGRLYYEGKWVPADAVKAEE  332
P. aeruginosa    QGDLEK---MLGYLKNAQD---------AAQ---PRAELLLGRLYYDGKWAPQDPRKAER  335

P. putida        HLQAAAEAGEISAHYYLGQLYRRGYLGNVEPQKAVDHLLAAARGGQNSADYALAQLFSEG  400
P. syringae      HLKKA-TATEISAHYYLGQIYRRGYLGQVYPQKAVDELLTAARGGQNSADFALAQLFSQG  391
P. aeruginosa    HLLKA-AASEPQANYYLGQIYRRGFLGKVYPQKAVDHLILAARAGQASADMALAQLWSQG  394

P. putida        HGIRPQPGNAWVFAQLSQANPTPQSAELLQQLDQQLTPDQRNQA----QQLLDQEKRARG  456
P. syringae      KGTLPDPVNAWVFAQLSLASETPQATELAQAMSTQLPPEKLATA----KDLLAREQKVRG  447
P. aeruginosa    RGIQPNRVNAYVFGQLAVQQQVPQASDLLGQIEAQLPPAERSQA----QQLLKREQQSRG  450
```

Supplementary Figure 5: Model of the *Pseudomonas syringae* and *Pseudomonas aeruginosa* AlgKX complex interaction interface. a, AlphaFold2 models of the *P. syringae* AlgKX (dark grey) and *P. aeruginosa* AlgKX (light grey) models superimposed with the crystal structure of *P. putida* AlgKX (periwinkle), with C_α RMSDs of 0.847 and 0.681 Å, respectively. **b**, Interaction interface of the AlphaFold2 *P. syringae* AlgKX model. **c**, Interaction interface of the AlphaFold2 *P. aeruginosa* AlgKX model. **d**, Multiple sequence alignment of AlgX regions involved in complex formation from *P. putida*, *P. syringae*, and *P. aeruginosa*. **e**, Multiple sequence alignment of AlgK regions involved in complex formation from *P. putida*, *P. syringae*, and *P. aeruginosa*. Bolded and highlighted residues indicate residues that are involved in the AlgKX interaction.

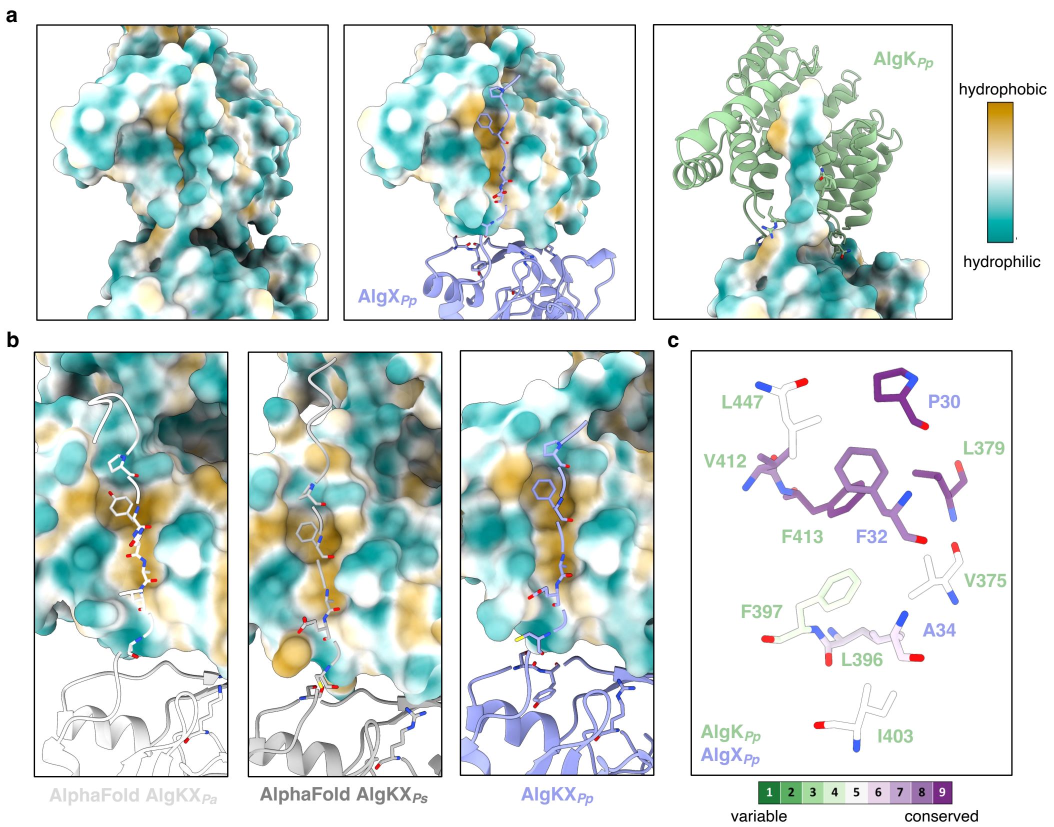

Supplementary Figure 6: Hydrophobic surface at AlgX N-terminus and AlgK groove. a, Hydrophobic surface representation of AlgKX$_{Pp}$ (left), AlgX$_{Pp}$ (middle), and AlgK$_{Pp}$ (right) in the AlgKX$_{Pp}$ complex. **b**, Hydrophobic surface representation of AlgK$_{Pa}$ in the AlphaFold AlgKX$_{Pa}$ model (left), AlgK$_{Ps}$ in the AlphaFold AlgKX$_{Ps}$ model (middle), and AlgK$_{Pp}$ in the AlgKX$_{Pp}$ structure (right). **c**, Sequence conservation of residues in the AlgKX$_{Pp}$ structure involved in the hydrophobic regions, as calculated by ConSurf[10]. Resides in AlgK$_{Pp}$ and AlgX$_{Pp}$ are labeled in light green and periwinkle, respectively.

a

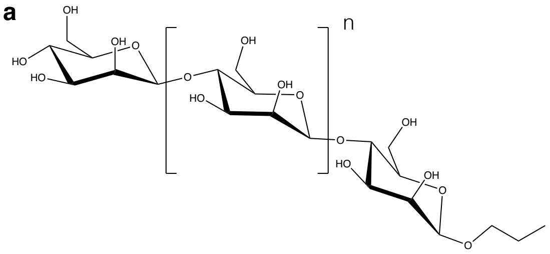

b ManA$_6$

n = 4
Chemical Formula: $C_{39}H_{56}O_{37}$
Exact Mass: 1116.25
Molecular Weight: 1116.84
m/z: 1116.25 (100.0%), 1117.25 (43.6%), 1118.26 (9.5%), 1118.25 (7.6%), 1119.26 (4.7%), 1120.26 (1.1%)
Elemental Analysis: C, 41.94; H, 5.05; O, 53.00

c ManA$_7$

n = 5
Chemical Formula: $C_{45}H_{64}O_{43}$
Exact Mass: 1292.28
Molecular Weight: 1292.96
m/z: 1292.28 (100.0%), 1293.29 (51.0%), 1294.29 (21.6%), 1295.29 (6.5%), 1296.29 (1.5%)
Elemental Analysis: C, 41.80; H, 4.99; O, 53.21

d ManA$_{10}$

n = 8
Chemical Formula: $C_{62}H_{88}O_{61}$
Exact Mass: 1820.38
Molecular Weight: 1821.34
m/z: 1820.38 (100.0%), 1821.38 (71.5%), 1822.30 (25.2%), 1823.39 (14.8%), 1822.38 (12.5%), 1824.30 (4.9%), 1825.39 (1.2%)
Elemental Analysis: C, 41.54; H, 4.87; O, 53.59

e ManA$_{11}$

n = 9
Chemical Formula: $C_{69}H_{96}O_{67}$
Exact Mass: 1996.41
Molecular Weight: 1997.46
m/z: 1996.41 (100.0%), 1997.41 (77.2%), 1998.42 (30.2%), 1999.42 (18.4%), 1998.41 (13.8%), 2000.42 (6.4%), 20001.42 (1.6%), 1997.42 (1.1%)
Elemental Analysis: C, 41.49; H, 4.84; O, 53.67

f ManA$_{12}$

n = 10
Chemical Formula: $C_{75}H_{104}O_{73}$
Exact Mass: 2172.44
Molecular Weight: 2173.58
m/z: 1996.41 (100.0%), 1997.41 (77.2%), 1998.42 (30.2%), 1999.42 (18.4%), 1998.41 (13.8%), 2000.42 (6.4%), 20001.42 (1.6%), 1997.42 (1.1%)
Elemental Analysis: C, 42.44; H, 4.82; O, 53.73

Supplementary Figure 7: Chemical structures of mannuronate oligosaccharides used in ESI-MS assay. a, General chemical structure of the mannuronate oligosaccharides used. **b**, ManA$_6$; **c**, ManA$_7$; **d**, ManA$_{10}$; **e**, ManA$_{11}$; **f**, ManA$_{12}$.

a GMGMGM

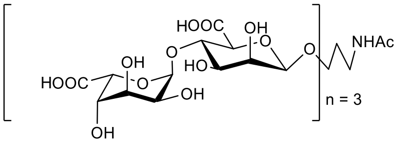

Chemical Formula: $C_{41}H_{59}NO_{38}$
Exact Mass: 1173.27
Molecular Weight: 1173.89
m/z: 1173.27 (100.0%); 1174.27 (44.7%); 1175.28 (18.4%); 1176.28 (5.2%); 1174.28 (2.1%); 1177.28 (1.2%)
Elemental Analysis: C, 41.95; H, 5.07; N, 1.19; O, 51.79

b GMGGMG

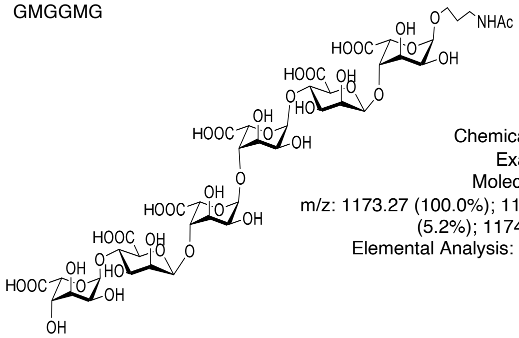

Chemical Formula: $C_{41}H_{59}NO_{38}$
Exact Mass: 1173.27
Molecular Weight: 1173.89
m/z: 1173.27 (100.0%); 1174.27 (44.7%); 1175.28 (18.4%); 1176.28 (5.2%); 1174.28 (2.1%); 1177.28 (1.2%)
Elemental Analysis: C, 41.95; H, 5.07; N, 1.19; O, 51.79

c GMGMGMG

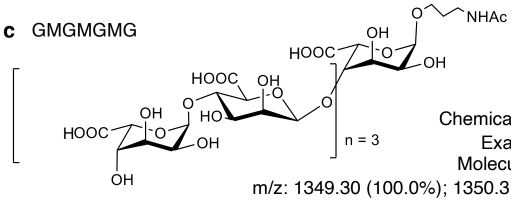

Chemical Formula: $C_{47}H_{67}NO_{44}$
Exact Mass: 1349.30
Molecular Weight: 1350.02
m/z: 1349.30 (100.0%); 1350.31 (53.3%); 1351.31 (23.0%); 1352.31 (7.1%); 1353.31 (1.6%)
Elemental Analysis: C, 41.81; H, 5.00; N, 1.04; O, 52.15

Supplementary Figure 8: Chemical structures of mannuronate-guluronate oligosaccharides used in ESI-MS assay. a, GMGMGM; **b**, GMGGMG; **c**, GMGMGMG.

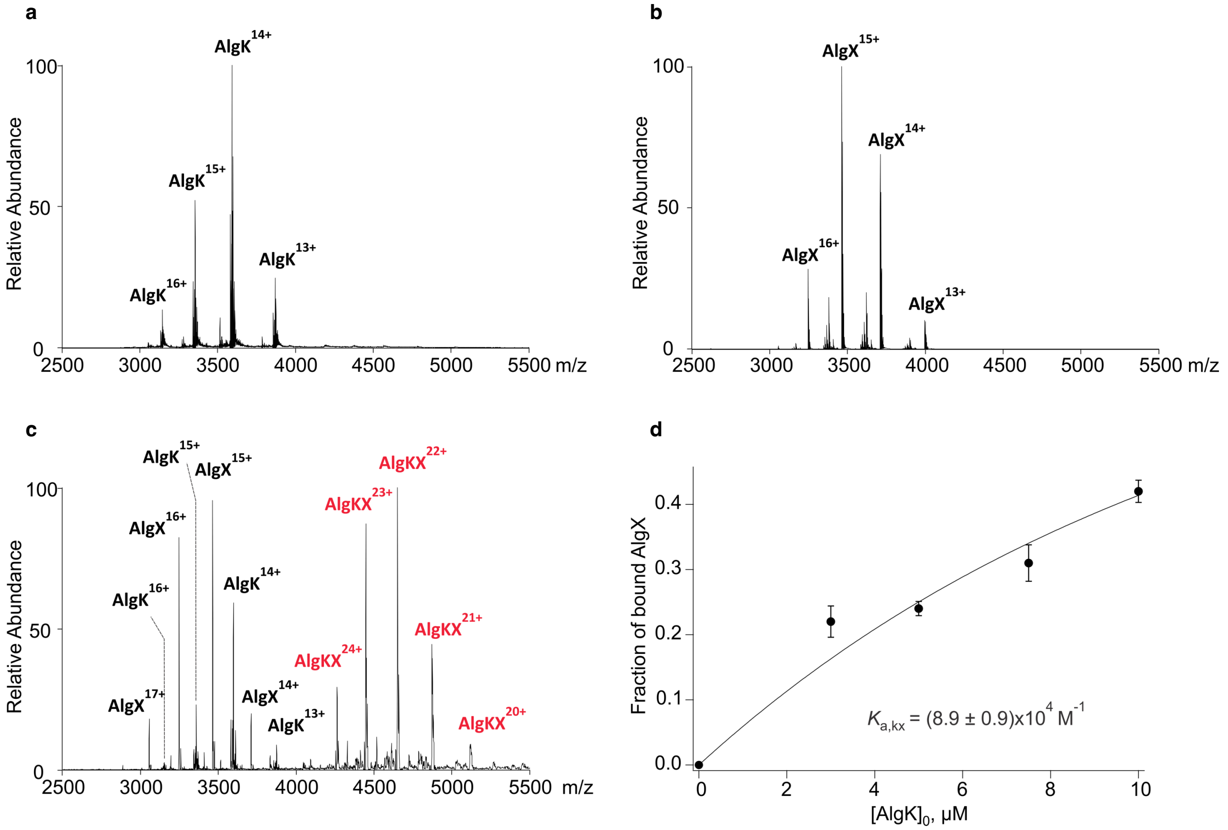

Supplementary Figure 9: Representative ESI mass spectra of AlgK$_{\boldsymbol{Pp}}$ (≡ AlgK), AlgX$_{\boldsymbol{Pp}}$ (≡ AlgX), and the AlgKX$_{\boldsymbol{Pp}}$ complex (≡ AlgKX). Representative ESI mass spectra acquired for ammonium acetate (200 mM, pH 7.0) solutions of **a**, AlgK$_{Pp}$ (5 µM); **b**, AlgX$_{Pp}$ (5 µM); **c**, AlgK$_{Pp}$ and AlgX$_{Pp}$ (5 µM each protein). **d**, Plot of fraction of AlgX$_{Pp}$ bound to AlgK$_{Pp}$ versus initial AlgK$_{Pp}$ concentration. Calculated association constant ($K_{\mathrm{a,kx}}$) value for 1:1 formation of the AlgKX$_{Pp}$ complex is shown on the plot. Three technical replicates were performed (n = 3). Values represent the mean +/- SD.

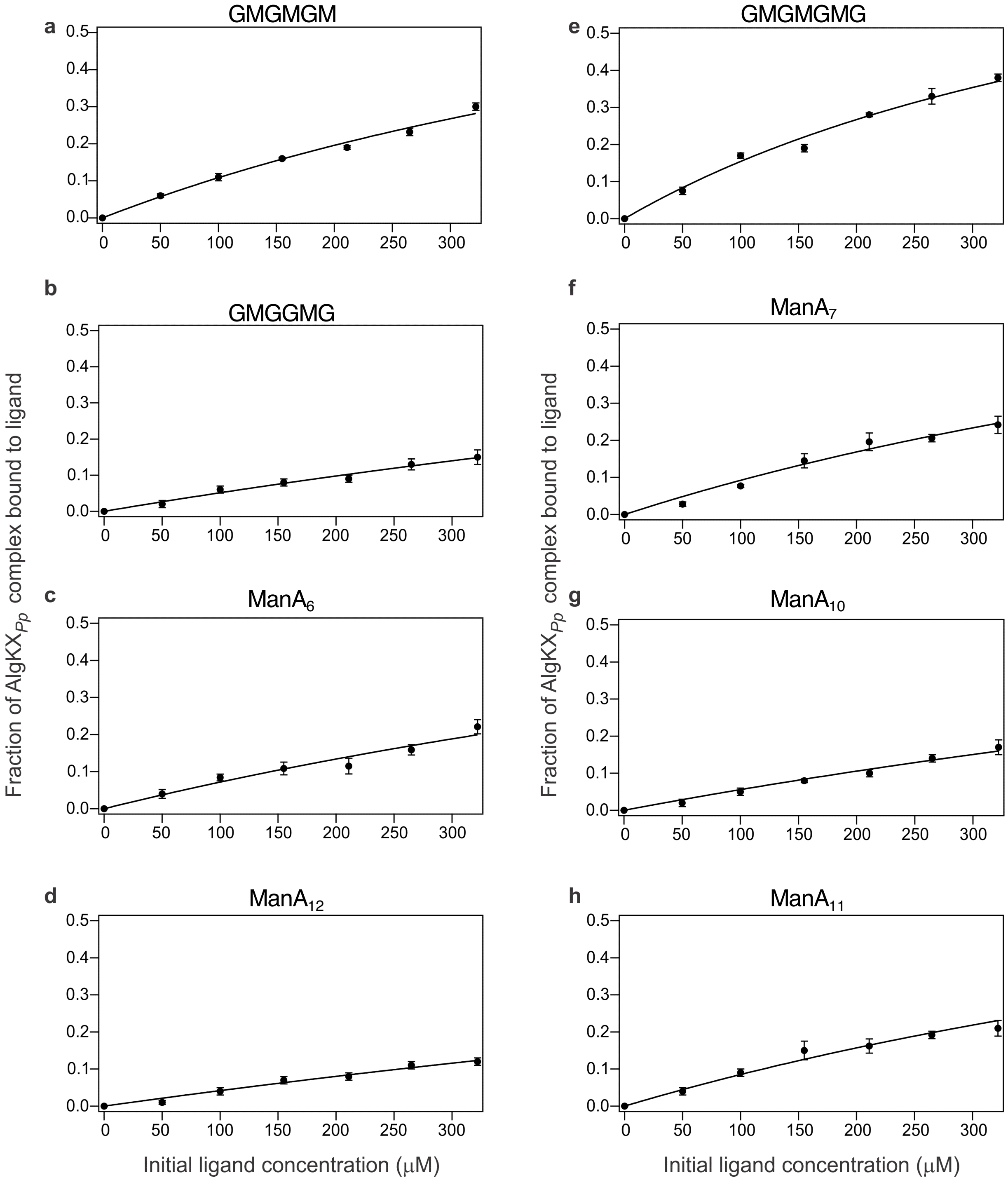

Supplementary Figure 10: Plot of fraction of AlgKX$_{Pp}$ bound to various alginate ligands in ammonium acetate (200 uM, pH 7.0) versus initial ligand concentration. a, GMGMGM; **b**, GMGGMG; **c**, ManA$_6$; **d**, ManA$_{12}$; **e**, GMGMGMG; **f**, ManA$_7$; **g**, ManA$_{10}$; **h**, ManA$_{11}$.Three technical replicates were performed for each ligand concentration (*n* = 3). Values represent the mean +/- SD.

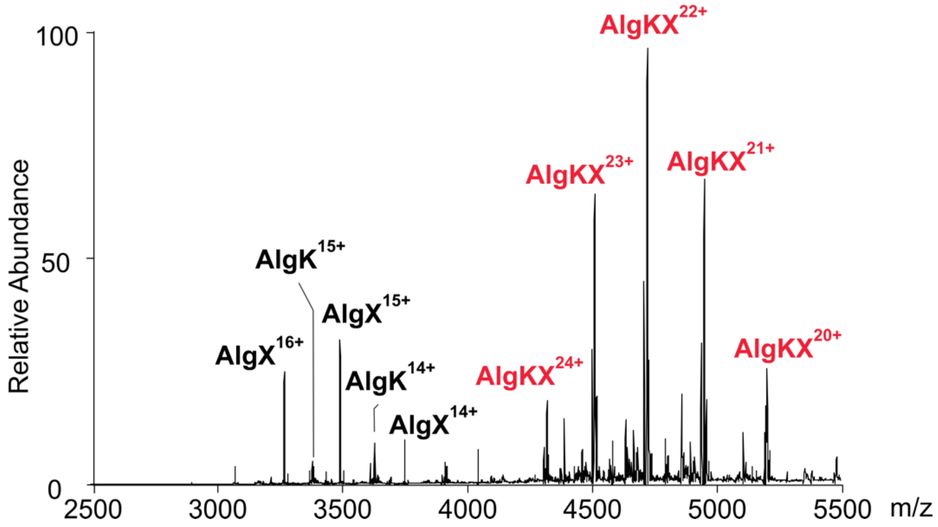

Supplementary Figure 11: Representative ESI mass spectra to examine hyaluronic acid ligand binding by the AlgKX*Pp* complex. ESI mass spectra acquired for ammonium acetate (200 mM), pH 7.0 solutions of AlgK*Pp* and AlgX*Pp* (5 µM each protein) with a 15-mer of hyaluronic acid (100 µM) demonstrates no specific binding.

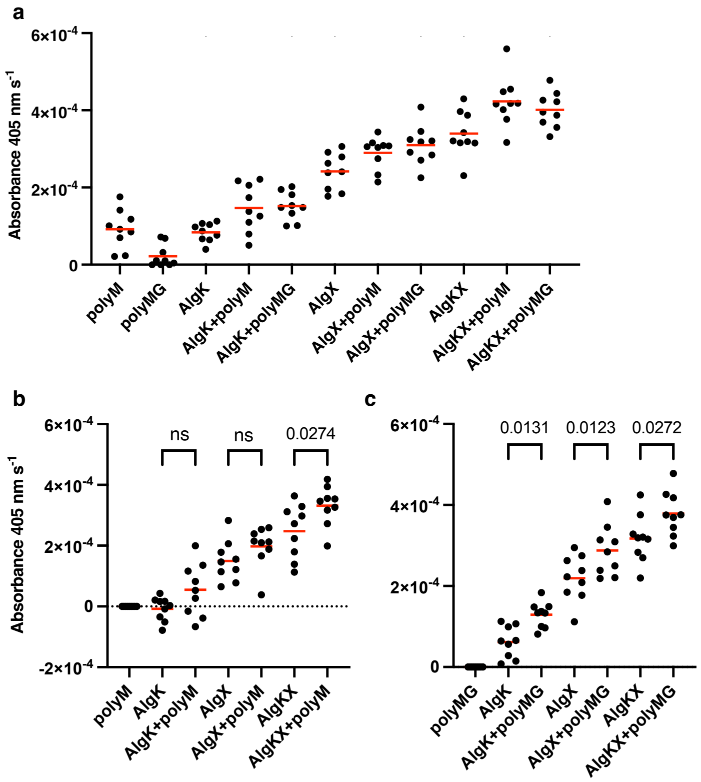

Supplementary Figure 12: Acetylesterase activity assay. a, Rate of acetylesterase activity measured by the absorbance value at 405 nm per second of the enzyme reaction using a *p*NP-acetate pseudosubstrate. Values represent three technical replicates across three separate experiments (n = 9). **b,** Baseline-corrected values from **a**. Baseline defined as polyM data. **c,** Baseline-corrected values from **a**. Baseline defined as polyMG data. Red lines represent the mean. Statistical analyses were carried out using a one-way analysis of variance with Bonferroni correction: ns indicates not significant, p values indicated directly on graphs.

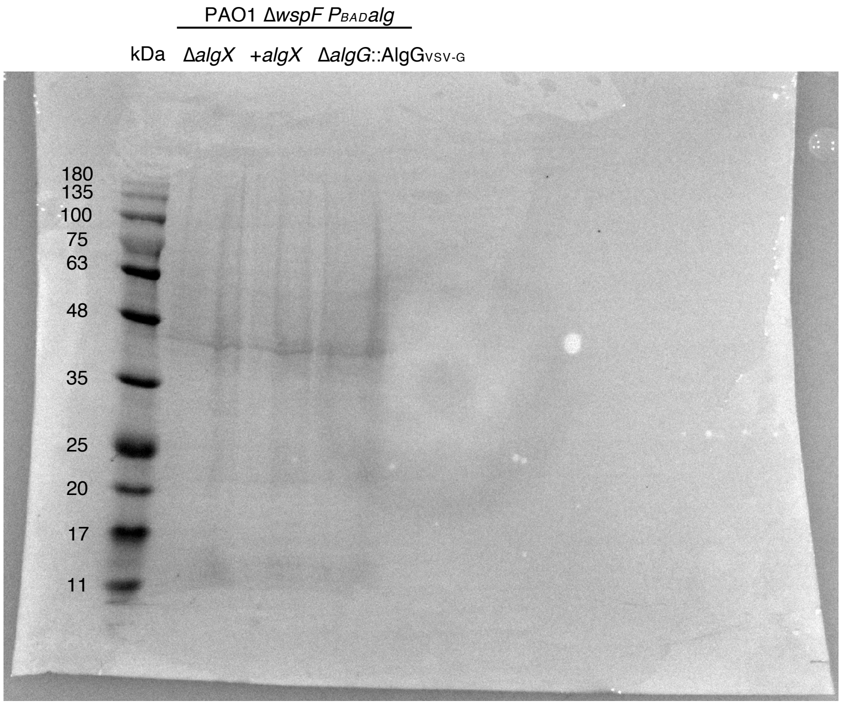

Supplementary Figure 13: Ponceau S stain of blot from Figure 4c demonstrating total protein loaded in each well. This experiment was repeated independently one other time.

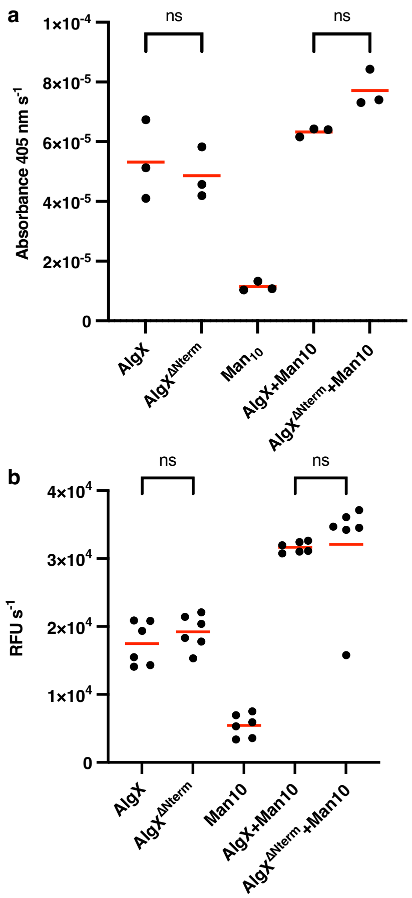

Supplementary Figure 14: AlgX and AlgX$^{\Delta Nterm}$ have comparable acetylesterase activities *in vitro*. **a**, Rate of acetylesterase activity measured by the absorbance value at 405 nm per second of the enzyme reaction using a *p*NP-acetate pseudosubstrate. Values represent three technical replicates across one experiment (n = 3). **b**, Rate of acetylesterase activity measured by fluorescence at 447 nm per second of the enzyme reaction using a 3-carboxyumbelliferyl acetate pseudosubstrate. Values represent three technical replicates across two separate experiments (n = 6). Man10 represents a nonacetylated polymannuronate substrate that is ten residues in length. Red lines represent the mean. Statistical analyses were carried out using a one-way analysis of variance with Bonferroni correction: ns indicates not significant.

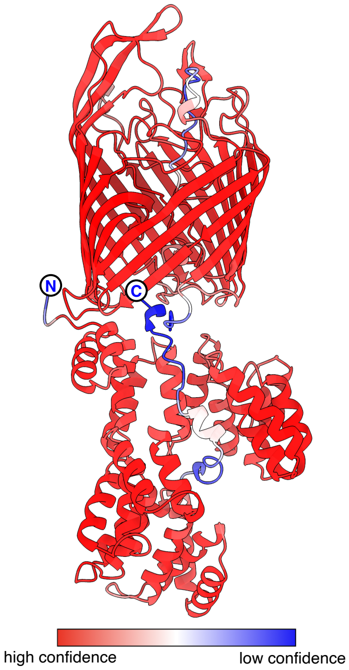

Supplementary Figure 15: Confidence score of the AlphaFold2 predicted AlgEK$_{Pp}$ model. AlgK$_{Pp}$ residues 243-280 were deleted to show the C-terminal coil (residues 447-484), which has an average confidence score of 56.94. Regions are coloured by confidence level (1-100); red represents high confidence (>90), blue represents low confidence (<50).

SUPPLEMENTARY REFERENCES

1. Chen, V. B. *et al.* MolProbity: All-atom structure validation for macromolecular crystallography. *Acta Crystallogr D Biol Crystallogr* **66**, 12–21 (2010).
2. Simon, R., Priefer, U. & Pühler, A. A Broad Host Range Mobilization System for *In Vivo* Genetic Engineering: Transposon Mutagenesis in Gram Negative Bacteria. *Bio/Technology* **1**, 784–791 (1983).
3. Franklin, M. J. *et al. Pseudomonas aeruginosa* AlgG is a polymer level alginate C5-mannuronan epimerase. *J Bacteriol* **176**, 1821–1830 (1994).
4. Ohman, D. E. & Chakrabarty, A. M. Genetic mapping of chromosomal determinants for the production of the exopolysaccharide alginate in a *Pseudomonas aeruginosa* cystic fibrosis isolate. *Infect Immun* **33**, 142–148 (1981).
5. Limoli, D. H. *et al. Pseudomonas aeruginosa* Alginate Overproduction Promotes Coexistence with *Staphylococcus aureus* in a Model of Cystic Fibrosis Respiratory Infection. *mBio* **8**, 1–18 (2017).
6. Gheorghita, A. A. *et al.* The *Pseudomonas aeruginosa* homeostasis enzyme AlgL clears the periplasmic space of accumulated alginate during polymer biosynthesis. *Journal of Biological Chemistry* **298**, 101560 (2022).
7. Hoang, T. T., Karkhoff-Schweizer, R. R., Kutchma, A. J. & Schweizer, H. P. A broad-host-range Flp-FRT recombination system for site-specific excision of chromosomally-located DNA sequences: application for isolation of unmarked *Pseudomonas aeruginosa* mutants. *Gene* **212**, 77–86 (1998).
8. Almblad, H. *et al.* The cyclic AMP-Vfr signaling pathway in *Pseudomonas aeruginosa* is inhibited by cyclic Di-GMP. *J Bacteriol* **197**, 2190–2200 (2015).
9. Choi, K. H. & Schweizer, H. P. mini-Tn7 insertion in bacteria with single attTn7 sites: Example *Pseudomonas aeruginosa*. *Nat Protoc* **1**, 153–161 (2006).
10. Ashkenazy, H. *et al.* ConSurf 2016: an improved methodology to estimate and visualize evolutionary conservation in macromolecules. *Nucleic Acids Res* **44**, W344–W350 (2016).
