# Supplementary material for: Structure of the AlgKX modification and secretion complex required for alginate production and biofilm attachment in Pseudomonas aeruginosa
Source: Nat Commun. 2022 Dec 9;13:7631. doi: 10.1038/s41467-022-35131-6 (PMC9734138; doi:10.1038/s41467-022-35131-6)
Supplement: Supplementary file 3 — Reporting Summary [file 41467_2022_35131_MOESM3_ESM.pdf]

nature portfolio

Corresponding author(s): P. Lynne Howell

Last updated by author(s): Nov 14, 2022

Reporting Summary

Nature Portfolio wishes to improve the reproducibility of the work that we publish. This form provides structure for consistency and transparency in reporting. For further information on Nature Portfolio policies, see our Editorial Policies and the Editorial Policy Checklist.

Statistics

For all statistical analyses, confirm that the following items are present in the figure legend, table legend, main text, or Methods section.

n/a	Confirmed	
☐	☒	The exact sample size (*n*) for each experimental group/condition, given as a discrete number and unit of measurement
☐	☒	A statement on whether measurements were taken from distinct samples or whether the same sample was measured repeatedly
☐	☒	The statistical test(s) used AND whether they are one- or two-sided<br>*Only common tests should be described solely by name; describe more complex techniques in the Methods section.*
☒	☐	A description of all covariates tested
☐	☒	A description of any assumptions or corrections, such as tests of normality and adjustment for multiple comparisons
☐	☒	A full description of the statistical parameters including central tendency (e.g. means) or other basic estimates (e.g. regression coefficient) AND variation (e.g. standard deviation) or associated estimates of uncertainty (e.g. confidence intervals)
☐	☒	For null hypothesis testing, the test statistic (e.g. F, t, r) with confidence intervals, effect sizes, degrees of freedom and P value noted<br>*Give P values as exact values whenever suitable.*
☒	☐	For Bayesian analysis, information on the choice of priors and Markov chain Monte Carlo settings
☒	☐	For hierarchical and complex designs, identification of the appropriate level for tests and full reporting of outcomes
☒	☐	Estimates of effect sizes (e.g. Cohen's d, Pearson's r), indicating how they were calculated

Our web collection on statistics for biologists contains articles on many of the points above.

Software and code

Policy information about availability of computer code

Data collection	X-ray diffraction data were collected on beamline CMCF-BM (08B1-1) at the Canadian Light Source using MxDC and MxLIVE for remote data collection; XDS version 20220110; PHENIX 1.20.1-4487 for PHENIX.REFINE and PHASER; Xcalibur (Thermo, Version 4.1)
Data analysis	TLSMD online server; ConSurf online server; Coot Version 0.9; GraphPad Prism 9; PyMol (The PyMOL Molecular Graphics System, Version 1.2r3pre, Schrödinger, LLC); ChimeraX 1.4 (Resource for Biocomputing Visualization, and Informatics RBVI, UCSF); MolProbity in PHENIX 1.20.1-4487; GraphPad Prism 9 (Dotmatics); AlphaFold 2 (Alphabet/Google DeepMind); SWARM software (https://github.com/pkitov/CUPRA-SWARM) November 26, 2021 version; Maple 2017 version (Maplesoft, Waterloo, Canada); Xcalibur (Thermo, Version 4.1);

For manuscripts utilizing custom algorithms or software that are central to the research but not yet described in published literature, software must be made available to editors and reviewers. We strongly encourage code deposition in a community repository (e.g. GitHub). See the Nature Portfolio guidelines for submitting code & software for further information.

Data

Policy information about availability of data

All manuscripts must include a data availability statement. This statement should provide the following information, where applicable:

- Accession codes, unique identifiers, or web links for publicly available datasets
- A description of any restrictions on data availability
- For clinical datasets or third party data, please ensure that the statement adheres to our policy

PDB (https://www.rcsb.org/)

The coordinates and structure factors for the AlgKX complex have been deposited in the PDB, code 7ULA, no restrictions. All data described are located within the manuscript and the supplemental information.

Human research participants

Policy information about studies involving human research participants and Sex and Gender in Research.

Reporting on sex and gender	N/A
Population characteristics	N/A
Recruitment	N/A
Ethics oversight	N/A

Note that full information on the approval of the study protocol must also be provided in the manuscript.

Field-specific reporting

Please select the one below that is the best fit for your research. If you are not sure, read the appropriate sections before making your selection.

- [x] Life sciences
- [ ] Behavioural & social sciences
- [ ] Ecological, evolutionary & environmental sciences

For a reference copy of the document with all sections, see nature.com/documents/nr-reporting-summary-flat.pdf

Life sciences study design

All studies must disclose on these points even when the disclosure is negative.

Sample size	Statistical methods were not employed to determine sample size. Sample size was chosen to be greater or equal to triplicate when possible, with multiple experiments. These sample sizes were sufficient as they are consistent with general practice for biochemical and protein work, or previously published studies. Crystal violet microtitre plate assay was done with 8,9,9 technical replicates, which is greater than the usual 3,3,3. Due to the amount of sample available and ease of setting up the experiment, multiple replicates were set up and all data was included in the analysis and final figure. There is precedence for such a high technical replicate number using this assay (George A. O'Toole. Microtitre Dish Biofilm Formation Assay. 2011. J Vis Exp. doi: 10.3791/2437).
Data exclusions	Data was not excluded.
Replication	In our hands, results were reproducible as shown by the data. Experiments that were or were not repeated more than once are indicated as stated in the legend for each experiment.
Randomization	This is not applicable. There were no participants that could be randomly assigned to groups or treatments. This study was in vitro experiments only using purified proteins and homogeneous populations of bacteria.
Blinding	This is not applicable. Blinding is not typical for structural and biochemical in vitro experiments and thus not blinded.

Reporting for specific materials, systems and methods

We require information from authors about some types of materials, experimental systems and methods used in many studies. Here, indicate whether each material, system or method listed is relevant to your study. If you are not sure if a list item applies to your research, read the appropriate section before selecting a response.

Materials & experimental systems

n/a	Involved in the study	
☐	☒	Antibodies
☒	☐	Eukaryotic cell lines
☒	☐	Palaeontology and archaeology
☒	☐	Animals and other organisms
☒	☐	Clinical data
☒	☐	Dual use research of concern

Methods

n/a	Involved in the study	
☒	☐	ChIP-seq
☒	☐	Flow cytometry
☒	☐	MRI-based neuroimaging

Antibodies

Antibodies used	Monoclonal antibody against bacterial RNA polymerase beta (rpoB) (clone 8RB13) was purchased from Invitrogen (RRID: AB_795355) (catalog # MA1-25425 purchased through ThermoFisher); Goat anti-Mouse IgG (H+L) Secondary Antibody, HRP from Bio-Rad (Conjugate #1706516); Goat Anti-Rabbit IgG (H+L)-HRP from Bio-Rad (Conjugate #1706515); anti-AlgL; anti-AlgX; anti-AlgK; anti-AlgG
Validation	For bacterial RNA polymerase beta antibody, Invitrogen states that "MA1-25425 detects RNA polymerase beta from E. coli. and many other prokaryotes samples". This antibody was used in a Western blot for RNA polymerase in Burkholderia thailandensis in the peer-reviewed study Lennings, J., Mayer, C., Makhlouf, M., Brotz-Oesterhelt, H., Schwarz, S. (2019) Polar Localization of the ATPase ClpV-5 occurs independent of type VI secretion system apparatus proteins in Burkholderia thailandensis. BMC Res Notes. 12. doi: 10.1186/s13104-019-4141-3.<br>For goat anti-mouse HRP, we have previously used this antibody in a dot blot in the peer-reviewed study Gheorghita, A. A., Wolfram, F., Whitfield, G. B., Jacobs, H. M., Pfoh, R., Wong, S. S. Y., Guitor, A. K., Goodyear, M. C., Berezuk, A. M., Khurisgara, C. M., Parsek, M. R., Howell, P. L. (2022) The Pseudomonas aeruginosa homeostasis enzyme AlgL clears the periplasmic space of accumuulated alginate during polymer biosynthesis. J Biol Chem. 286. 101560.<br>For goat anti-rabbit HRP, we have previously used this antibody in Western blots in the peer-reviewed study Gheorghita, A. A., Wolfram, F., Whitfield, G. B., Jacobs, H. M., Pfoh, R., Wong, S. S. Y., Guitor, A. K., Goodyear, M. C., Berezuk, A. M., Khurisgara, C. M., Parsek, M. R., Howell, P. L. (2022) The Pseudomonas aeruginosa homeostasis enzyme AlgL clears the periplasmic space of accumuulated alginate during polymer biosynthesis. J Biol Chem. 286. 101560.<br>For anti-AlgL; we have previously used this antibody in Western blots in the peer-reviewed study Gheorghita, A. A., Wolfram, F., Whitfield, G. B., Jacobs, H. M., Pfoh, R., Wong, S. S. Y., Guitor, A. K., Goodyear, M. C., Berezuk, A. M., Khurisgara, C. M., Parsek, M. R., Howell, P. L. (2022) The Pseudomonas aeruginosa homeostasis enzyme AlgL clears the periplasmic space of accumuulated alginate during polymer biosynthesis. J Biol Chem. 286. 101560.<br>For anti-AlgX, anti-AlgK, and anti-AlgG, these antibodies are used routinely in our lab and our present study provides adequate proof of validation.
